# Geographical variations, prevalence, and molecular dynamics of fastidious phloem-limited pathogens infecting sugar beet across Central Europe

**DOI:** 10.1371/journal.pone.0306136

**Published:** 2024-07-02

**Authors:** Bojan Duduk, Jelena Stepanović, Jana Fránová, Agnieszka Zwolińska, Emil Rekanović, Miloš Stepanović, Nina Vučković, Nataša Duduk, Ivana Vico

**Affiliations:** 1 Institute of Pesticides and Environmental Protection, Belgrade, Serbia; 2 Biology Centre, Czech Academy of Sciences, Institute of Plant Molecular Biology, Plant Virology, Ceske Budejovice, Czech Republic; 3 Department of Plant Physiology, Faculty of Biology, Adam Mickiewicz University, Poznań, Poland; 4 University of Belgrade–Faculty of Agriculture, Belgrade, Serbia; CPRI: Central Potato Research Institute, INDIA

## Abstract

In Europe, two fastidious phloem-limited pathogens, ’*Candidatus* Phytoplasma solani’ (16SrXII-A) and ’*Candidatus* Arsenophonus phytopathogenicus’, are associated with rubbery taproot disease (RTD) and syndrome basses richesses (SBR) of sugar beet, respectively. Both diseases can significantly reduce yield, especially when accompanied by root rot fungi. This study investigates the presence, geographic distribution and genetic traits of fastidious pathogens and the accompanying fungus, *Macrophomina phaseolina*, found on sugar beet across four geographically separated plains spanning seven countries in Central Europe. The survey revealed variable incidences of symptoms linked to these fastidious pathogens in the Pannonian and Wallachian Plains, sporadic occurrence in the North European Plain, and no symptomatic sugar beet in the Bohemian Plain. Molecular analyses unveiled the occurrence of both ’*Ca*. P. solani’ and ’*Ca*. A. phytopathogenicus’ throughout Central Europe, with a predominance of the phytoplasma. These fastidious pathogens were detected in all six countries surveyed within the Pannonian and Wallachian Plains, with only a limited presence of various phytoplasmas was found in the North European Plain, while no fastidious pathogens were detected in Bohemia, aligning with observed symptoms. While 16S rDNA sequences of ’*Ca*. P. solani’ remained highly conserved, multi-locus characterization of two more variable loci (*tuf* and *stamp*) unveiled distinct variability patterns across the plains. Notably, the surprising lack of variability of *tuf* and *stamp* loci within Central Europe, particularly the Pannonian Plain, contrasted their high variability in Eastern and Western Europe, corresponding to epidemic and sporadic occurrence, respectively. The current study provides valuable insights into the genetic dynamics of ’*Ca*. P. solani’ in Central Europe, and novel findings of the presence of ’*Ca*. A. phytopathogenicus’ in five countries (Slovakia, Czech Republic, Austria, Serbia, and Romania) and *M*. *phaseolina* in sugar beet in Slovakia. These findings emphasize the need for further investigation of vector–pathogen(s)–plant host interactions and ecological drivers of disease outbreaks.

## Introduction

Sugar beet (*Beta vulgaris*), the exclusive sugar source in Europe, is cultivated on approximately 3 Mha of land on the continent. While France and Germany stand as the leading producers of sugar beet, accounting for approximately 500,000 ha each, other Central European countries collectively cultivate sugar beet on approximately 450,000 ha (Faostat 2024). Even if the area of production is high, the sugar beet yield is affected by different biotic and abiotic factors. With regard to biotic factors, two diseases of European sugar beet are associated with fastidious, phloem-limited bacterial pathogens. The "basses richesses" syndrome (SBR) is associated with ’*Candidatus* Arsenophonus phytopathogenicus’, while rubbery taproot disease (RTD) is associated with ’*Candidatus* Phytoplasma solani’ 16SrXII-A (stolbur phytoplasma).

Symptoms of SBR in sugar beet include brownish discoloration of taproot vascular tissue, as well as deformation (asymmetry) and discoloration of both young and old leaves. The most prominent manifestation of SBR is reduction of sugar content in the taproots, from which the disease name originated [1]. Conversely, typical symptoms of RTD manifest in the latter half of July, beginning with leaf turgor loss during peak daytime heat. Subsequent stages of the disease involve yellowing and necrosis of the oldest leaves, starting at the leaf margins and progressing to complete plant decline. SBR has been described in France, Hungary, Germany, and Switzerland [2–5]. RTD significantly hampers sugar beet production in Serbia and some other parts of the Pannonian plain, whereas in France and Germany the phytoplasma has been reported as a minor pathogen [2, 6, 7]. However, a recent finding identified a novel phytoplasma strain, 16SrXII-P, which is related to, yet distinct from ’*Ca*. P. solani’– 16SrXII-A, the dominant strain in sugar beet in some regions of eastern Germany [8].

Though RTD-affected plants often exhibit wilting and a rubbery texture of taproots, they remain free of rot symptoms until the decline of the plant. However, despite the absence of initial rot symptoms either during or after the decline of the aboveground parts of the plant, taproots begin to deteriorate and rot–in Serbia this is predominantly due to charcoal root rot associated with *Macrophomina phaseolina*. Consequently, some taproots undergo complete rotting before reaching the harvest stage or after harvest at the clamps on the sides of the fields [7, 9]. Though certain symptoms are distinctive for each of the two diseases, there are common symptoms shared between plants affected with SBR and RTD, such as the yellowing and proliferation of young leaves. Moreover, the extent of symptom development is contingent upon time elapsed since infection, climate conditions and, notably, specific varieties of sugar beet. The shared symptoms and their varying levels of expression, as well as the occasional presence of mixed infection, may present a challenge in distinguishing between the two diseases in the field, emphasizing the importance of molecular diagnostics. A notable instance of this challenge was documented in our previous research conducted in East Germany (Saxony-Anhalt) [8]. In this region, two pathogens, specifically ’*Ca*. A. phytopathogenicus’ and a ’*Ca*. P. solani’-related phytoplasma strain 16SrXII-P, coexisted with differing prevalence. However, despite their distinct prevalence rates, no differentiation of symptoms was possible.

Known for its broad host range, *M*. *phaseolina* is a fungus that primarily infects plants under severe stress conditions. While sugar beet is not typically a target for *M*. *phaseolina*, infection by ’*Ca*. P. solani’, the agent causing RTD, may render sugar beet susceptible to *M*. *phaseolina*, resulting in a complex disease called charcoal root rot. Our previous study demonstrated that *M*. *phaseolina* almost exclusively attacks sugar beet that has been previously infected with ’*Ca*. P. solani’. Charcoal root rot exacerbates losses initially caused by infection of sugar beet with ’*Ca*. P. solani’ alone, i.e. RTD, potentially signifying a pivotal shift in the harmfulness of RTD, as demonstrated by devastating sugar beet yield losses in Serbia [9].

Both SBR and RTD represent significant economic concerns. The negative impact of SBR is primarily attributed to reduced sugar content in the taproot of sugar beet, leading to substantial economic losses. For instance, in France in 1991, economic losses exceeded 50% [1]. Yet quantifying the yield reduction linked to RTD proves challenging due to infield variability in distribution and severity of the disease. Nevertheless, substantial yield losses, including instances of up to 100%, were reported in 2023, particularly in Serbia where approximately 1500 ha of sugar beet were abandoned and plowed, rather than harvested (National Agricultural Advisory Service of Serbia and Saša Rajačić, Sunoko d.o.o., Novi Sad, Serbia, personal communications).

Information on the occurrence, genetic diversity, and geographical distribution of these fastidious pathogens of sugar beet in Europe is limited and available only for some regions. While extended surveys conducted in France, Germany, and Serbia have revealed the existence and distribution of these fastidious pathogens, other parts of Central Europe have not been thoroughly studied and thus only limited information is available for the latter [6, 7, 10–12]. Furthermore, no information about the presence of charcoal root rot of sugar beet and associated *M*. *phaseolina* is available in countries beyond Serbia. Given the varied scenarios regarding the distribution and prevalence of the fastidious pathogens reported in France, Germany, and Serbia, with each country displaying a distinct epidemiology, conducting surveys to ascertain the occurrence and prevalence of fastidious pathogens in individual growing regions is imperative. This is crucial for comprehending the issue and subsequently implementing appropriate control measures. The objectives of this study were therefore to (i) assess the presence of fastidious pathogen(s) in sugar beet in Central Europe, and then identify and molecularly characterize the pathogen(s) in relation to published and available sugar beet strains from other parts of Europe, and (ii) probe for the presence of charcoal root rot on RTD-affected fields, and isolate, identify and molecularly characterize *M*. *phaseolina* in relation to strains previously described in RTD-affected sugar beets in Serbia.

## Materials and methods

### Ethics statement

Our field survey was conducted in areas of commercial sugar beet production, which did not necessitate any specific permissions. The precise locations of our study area are detailed in Table 2. We confirm that our field study did not involve human participants, nor did it involve any endangered or protected species.

### Surveyed areas and sample collection

**Fig 1:**
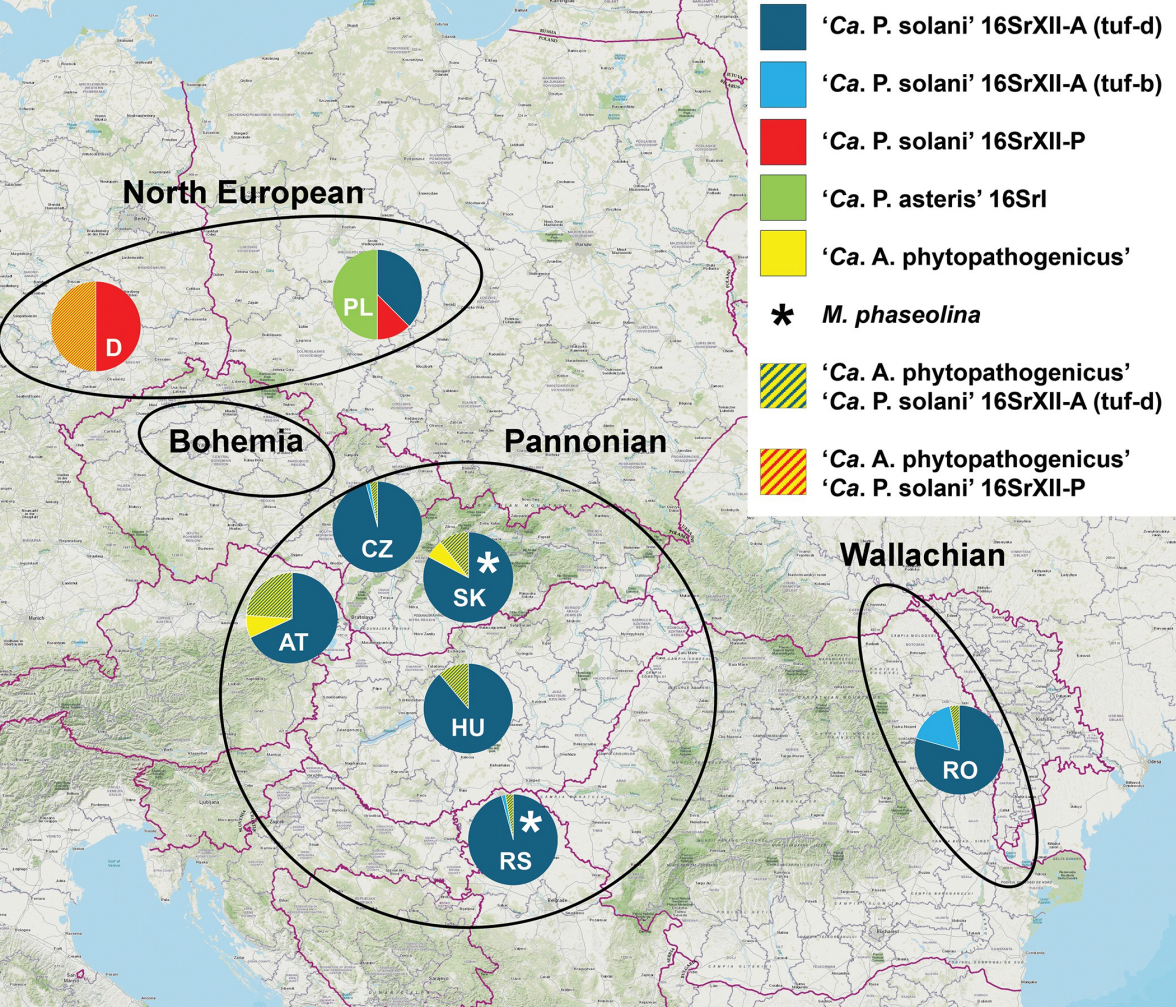
Distribution of fastidious sugar beet pathogens in Central Europe. Map of Central Europe depicts assessed plains and the proportion of evaluated fastidious pathogens of sugar beet per country. Chart sections represent the proportion of each specific fastidious pathogen/strain according to the specified color pattern provided in the legend on the right. The in-chart asterisks indicate the presence of *M*. *phaseolina*, whereas letters represent the country acronym. While results from Germany have been published previously (8), results from other countries were obtained in this study.

The surveys were conducted during October and November 2023 in seven major sugar beet-cultivating countries in Central Europe, excluding Germany. The surveyed area spanned the Pannonian Plain (Northern Serbia, Hungary, Lower Austria, Slovakia, and Moravia in the Czech Republic), and also encompassed Romanian Moldova (Wallachian Plain) in the east, as well as Bohemia in the Czech Republic and the Odra basin (North European Plain) in Poland in the north (Fig 1). Notably, the surveyed areas covered four geographically separated plains, namely the Wallachian, Pannonian, and Bohemian Plains, and parts of the North European Plain. In October, production sugar beet fields were extensively surveyed for RTD- and SBR-like symptoms in all main sugar beet-growing regions of Slovakia (five areas) and the Czech Republic (nine areas), as well as in 31 localities within two regions of Poland, namely Lower Silesia and Greater Poland. Furthermore, as part of their regular annual monitoring, the National Agricultural Advisory Service of Serbia and SESVanderHave SRB surveyed one and three areas, respectively, in all three sugar beet-growing regions of Serbia. AGRANA Research & Innovation Center conducted surveys in Romania (five areas) and Austria (six areas) within their respective production fields, while Beta Research Institute Nonprofit Ltd. Sopronhorpács carried out the survey in Hungary (four areas).

The on-site determination of typical RTD and SBR symptoms involved visual assessments of leaves, cross-cut examinations of taproots, and an evaluation of taproot flexibility. Due to the difficulty in distinguishing between the two diseases in the field, plants exhibiting symptoms that could be attributed to either of the two diseases were classified as RTD/SBR-symptomatic. Disease incidence (Table 1) was estimated by counting symptomatic plants in the most affected part of the field, as described previously [6] In locations where plants with typical RTD/SBR symptoms were observed, up to 40 symptomatic samples were collected from each locality, along with five asymptomatic samples as negative controls. In areas without specific RTD/SBR symptoms, sugar beets exhibiting nonspecific symptoms (attributable to RTD/SBR, as well as other pathogens or disorders) were collected. A total of 432 sugar beet samples with typical RTD/SBR symptoms were collected from seven surveyed countries, and were subsequently subjected to phytoplasma and ’*Ca*. A. phytopathogenicus’ assessment. Moreover, sugar beet samples with aspecific disorders were collected from localities in which typical RTD/SBR symptoms were not observed: 62 samples in four localities in Bohemia (CZ) and 68 samples in six localities in Poland. Sugar beet plants from Poland, the Czech Republic, Slovakia and Serbia were examined for root rot in addition to RTD/SBR symptoms. Additional 30 RTD/SBR-symptomatic samples from a field in Berny-en-Santerre, France (Western Europe), and 20 rubbery taproots from a clamp in Ust-Labinsk, Russia (Eastern Europe), were collected and included in phytoplasma and ’*Ca*. A. phytopathogenicus’ assessment for comparison, thus complementing the samples from Central Europe.

**Table 1:** Presence of the fastidious pathogens in sugar beet in Central Europe.

Locality	Symptom Prevalence*	RTD	SBR	Mixed
(number of collected samples)				
Piotrowice Świdnickie-PL (16)	<0.01%	3	0	0
Żołędnica-PL (27)	<0.01%	1**	0	0
Węgierskie -PL (11)	~5%	4**	0	0
Prušánky-CZ (27)	~100%	25	2	2
Lednice-CZ (19)	<0.1%	15	0	0
Litobratřice-CZ (13)	<0.1%	13	0	0
Břežany-CZ (15)	<0.1%	15	0	0
Sereď-SK (35)	~65%	25	5	3
Špačince-SK (10)	~0.1%	5	0	0
Považany-SK (10)	~0.1%	9	0	0
Rastislavice-SK (11)	~50%	10	4	4
**Kamenín-SK (22)**	~100%	16	4	1
Tirzii, Vaslui-RO (10)	N.A.	9	1	1
Dângeni, Botoşani-RO (10)	N.A.	2	0	0
Pașcani, Iași-RO (10)	N.A.	3	0	0
Hănești, Botoşani-RO (10)	N.A.	3	0	0
Bumbata, Vaslui-RO (10)	N.A.	6	0	0
Groβkrut-AT (10)	~50%	8	0	0
Pamhagen 1-AT (10)	~0.1%	5	2	0
Pamhagen 2-AT (10)	~0.1%	10	4	4
Pamhagen 3-AT (10)	~0.1%	1	5	1
Pamhagen 4-AT (10)	~50%	9	10	9
Wallern im Burgenland-AT (10)	~50%	10	6	6
Andau-AT (10)	~0.1%	9	2	1
Hollern-AT (10)	~50%	7	1	1
Sommerein-AT (10)	~0.1%	2	0	0
Cegléd-HU (12)	~5%	9	0	0
Nadudvar-HU (12)	~25%	12	5	5
Szentes-HU (12)	~30%	12	0	0
Szikancs-HU (12)	~100%	7	0	0
**Temerin-RS (40)**	~60%	37	2	2
**Botoš-RS (13)**	~70%	11	0	0
**Sremska Mitrovica-RS (14)**	~90%	12	0	0
**Mol-RS (4)**	~60%	4	0	0

*Symptom prevalence in the most severely affected part of the field; RTD–Number of samples in which RTD-associated ’*Ca*. P. solani’ (16SrXII-A) was detected

**with the exception of Żołędnica and Węgierskie, where 16SrXII-P and 16SrI phytoplasma were detected, respectively; SBR–Number of samples in which SBR-associated ’*Ca*. A. phytopathogenicus’ was detected; localities in which *M*. *phaseolina* was found are labeled in bold; N.A.–Not available.

### Nucleic acid extraction

For assessment of phloem-limited prokaryotes, nucleic acid was extracted from 0.5 g of taproot tissue of all sugar beet samples, according to the CTAB protocol [13]. Total nucleic acids were precipitated with isopropanol, resuspended in TE buffer (10 mM Tris pH 8 and 1 mM EDTA) and stored at -20˚C.

### Phytoplasma assessment

Nested PCR was employed for universal phytoplasma detection in all samples. Direct PCR assays were conducted using P1/P7 primers [14, 15] followed by nested PCR with R16F2n/R2 primers under previously described conditions [16]. Each 25 μL PCR mix composition was as previously described [8]. ’*Ca*. P. solani’ strain 284/09 [17] was used as a positive control, while reaction mixes lacking template DNA were employed as negative controls. In nested PCR, 0.5 μL of direct PCR amplicon was used as template DNA. Six microliters of PCR products were separated in a 1% agarose gel, stained with ethidium bromide and visualized with a UV transilluminator. Amplification of the fragment of expected size, ~1.2 Kbp, was considered a positive reaction. Identification of detected phytoplasmas was performed with RFLP analysis using the *Tru1*I (Thermo Fisher Scientific, Vilnius, Lithuania) restriction enzyme on R16F2n/R2 amplicons. Restriction products were separated in an 8% polyacrylamide gel, stained and visualized as described above.

Three ’*Ca*. P. solani’-positive samples, identified as 16SrXII-A, were randomly selected per locality (where possible) for further multilocus sequence analysis (MLSA). In addition to 16S rDNA, the *tuf* and *stamp* gene sequences were analyzed in selected samples. The *tuf* gene was amplified using the Tuf1-f1/r1 primer pair, followed by fTufAy/rTufAy in nested PCR assays [9, 18, 19]https://apsjournals.apsnet.org/doi/10.1094/PDIS-07-20-1602-RE - b44. For amplification of the *stamp* gene, nested PCR assays were conducted using the Stamp-F/R0 primer pair, followed by Stamp-F1/R1 [20]. All PCR products were sequenced in both directions using the primers previously applied in their amplification, whereas P1/P7 amplicons were additionally sequenced using primers 350R, M1 (758F), R16(I)R1 and B6 (16R758F) [21–23] to cover near full-length 16S rRNA gene, which was performed by a commercial service (Macrogen Inc., Seoul, Korea). The obtained 16S rDNA, *tuf* and *stamp* sequences were assembled with Pregap4 from the Staden Package [24], manually inspected and aligned with publicly available sequences of various strains of ’*Ca*. P. solani’ genotypes [7, 25–28] using ClustalX, under MEGA version X [29, 30]. The obtained 16S rDNA/*tuf*/*stamp* sequences of strains RU106/23, RU122/23, FR662/23 and FR665/23 were deposited in GenBank under accession numbers PP716917/PP731983/PP731984, PP716577/PP731985/PP731986, PP716918/PP731987/PP731988 and PP716578/PP731989/PP731990, respectively. Evolutionary history based on the *stamp*-deduced amino acid sequences of ’*Ca*. P. solani’ 16SrXII-A strains from sugar beet, obtained in the current and previous studies [6, 7, 31], was inferred using the Maximum-Likelihood (ML) method (MEGA X) and Jones-Taylor-Thornton (JTT) model [32] using a discrete Gamma distribution as the best-fit substitution model. Initial trees for the heuristic search were obtained automatically by applying the Neighbor-Join and BioNJ algorithms. To estimate the statistical significance of the inferred clades, 1,000 bootstraps were performed.

In addition to 16S rDNA, the *tuf*, *secY*, and *rp* genes of the sample identified as 16SrXII-P were analyzed. The *tuf* gene was amplified as described for 16SrXII-A strains. To amplify *secY* and *rp* genes, L15F1/MapR1 followed by AYsecYF1/AYsecYR1 and rpL2F3/rp(I)R1A primer pairs were used in nested and direct PCR assays, respectively [33, 34]. The PCR mix composition for all reactions, as well as PCR conditions, were as previously described [18, 20, 33, 34]. PCR products obtained by amplifying 16S rRNA (P1/P7), *tuf* (Tuf1-f1/Tuf1-r1), *secY* (AYsecYF1/AYsecYR1), and *rp* (rpL2F3/rp(I)R1A) genes were sequenced in both directions. The reads were assembled and inspected as described above, and the 16S rRNA, *tuf*, *secY*, and *rp* gene sequences of strain PL359/23 were deposited in GenBank under accession numbers PP716579, PP731991, PP7319913and PP731992, respectively.

### Assessment of ’*Ca*. A. phytopathogenicus’

The presence of ’*Ca*. A. phytopathogenicus’ in all samples was assessed using the TaqMan real-time PCR (qPCR) protocol to target the *hsp20* gene (small heat shock protein), with previously described cycling parameters and reaction mix [12]. A DNA-free blank assay was included as a negative control corresponding to an asymptomatic sugar beet, whereas strain HN1220/5 [6] was used as a positive control for ’*Ca*. A. phytopathogenicus’. All qPCRs were performed in the Magnetic Induction Cycler, Mic (Bio Molecular Systems, Upper Coomera, Australia). Data evaluation for all qPCR assays was conducted using micPCR^©^ software Version 2.6.4 (Bio Molecular Systems, Upper Coomera, Australia). Samples were considered positive if they produced an amplification curve with exponential growth and a C_q_ value of <40 [12]. For confirmation of obtained results, all samples that tested positive for ’*Ca*. A. phytopathogenicus’ in the qPCR assay underwent additional separate PCR reactions using the Alb1/Oliv1 and Fra5/4 primer pairs, which target the internal transcriber spacer region between the 16S and 23S ribosomal genes, and the 16S rRNA gene of ’*Ca*. A. phytopathogenicus’, respectively. The reaction mix and conditions were consistent with those described previously [35, 36]. A positive reaction for the Alb1/Oliv1 assay was indicated by the amplification of four fragments ranging from 500–1000 bp, while for the Fra5/4 assay, a positive reaction was indicated by the amplification of a fragment of approximately 550 bp. Samples that tested negative in the PCR with Fra5/4 primers, but were positive in the qPCR assay, were then subjected to a semi-nested PCR assay in which 27F/Fra4 primers [37] were used in the direct reaction, and Fra5/4 primers were used in the nested reaction to increase sensitivity. Three Fra5/4 PCR-positive samples from each area were sequenced in both directions using the primers applied for amplification, yielding 510-nt sequences of the ’*Ca*. A. phytopathogenicus’ partial 16S rRNA gene. These sequences were assembled, manually inspected, and compared with publicly available sequences as described above.

### Assessment of *Macrophomina phaseolina*

Sugar beet samples from the Czech Republic, Slovakia and Serbia that showed both root rot and RTD/SBR symptoms were tested for the presence of fungi, in addition to the fastidious pathogens. Fungi were isolated from the margin of healthy and rotted tissue. Root fragments were washed, disinfected in 70% ethanol and placed on potato dextrose agar (PDA, EMD, Darmstadt, Germany, pH 5.6 ± 0.2) in Petri dishes (90 mm). After 3–5 days of incubation at 24 ± 2°C in 12/12 h light/dark regime, developing fungal colonies were transferred to a pure culture and their morphology was assessed. Isolates with colony features typical for *Macrophomina* sp. (initially whitish colonies that become dark grey with age and develop numerous black sclerotia) [38] were further subjected to molecular analyses for fungal species confirmation.

Fungal DNA was extracted from 7-day-old cultures of isolates, according to the previously described CTAB protocol [39]. The isolates were tested using *M*. *phaseolina*-specific primers for amplification of translation elongation factor 1α (*TEF1*-α) MpTefF/MpTefR, following previously described conditions [40]. Amplification of the fragment of expected size, ~220 bp, was considered a positive reaction. All *M*. *phaseolina* isolates were subjected to further molecular characterization based on *TEF1*-α, using primer pair EF1-728F/EF2R [41, 42]. Nine isolates from Serbia (four, four and one per *TEF1*-α haplotype) and all eight from Slovakia were selected for MLSA. In addition to *TEF1*-α, four other loci (internal transcribed spacer regions 1 and 2 including the 5.8S rRNA gene (ITS), actin (*ACT*), calmodulin (*CAL*), and β-tubulin (*TUB*) genes) were analyzed. These loci were amplified using the following primer pairs: ITS1/ITS4 [43], ACT-512F/ACT-783R [42], CAL-228F/CAL-737R [42], and T1/Bt2b [44, 45], respectively. The PCR conditions and reaction mix applied for amplification of these loci were described previously [9]. Obtained PCR products were then separated and visualized as described above. Amplified products were sequenced in both directions; sequences were assembled, inspected and compared with those publicly available as described above. The accession numbers of sequences deposited in GenBank are provided in S1 Table. Evolutionary history was inferred based on combined analyses of the five loci (ITS, *TEF1*-α, *ACT*, *CAL*, and *TUB*) of 17 isolates obtained in this study and reference isolates of *Macrophomina* spp. (S1 Table), using the ML method (MEGA X) and Hasegawa–Kishino–Yano (HKY) model [46] as the best-fit substitution model. Initial tree(s) for the heuristic search were obtained automatically by applying Neighbor-Join and BioNJ algorithms. To estimate the statistical significance of the inferred clades, 1,000 bootstraps were performed.

## Results

### Field status of RTD/SBR symptoms and their geographic distribution

**Fig 2:**
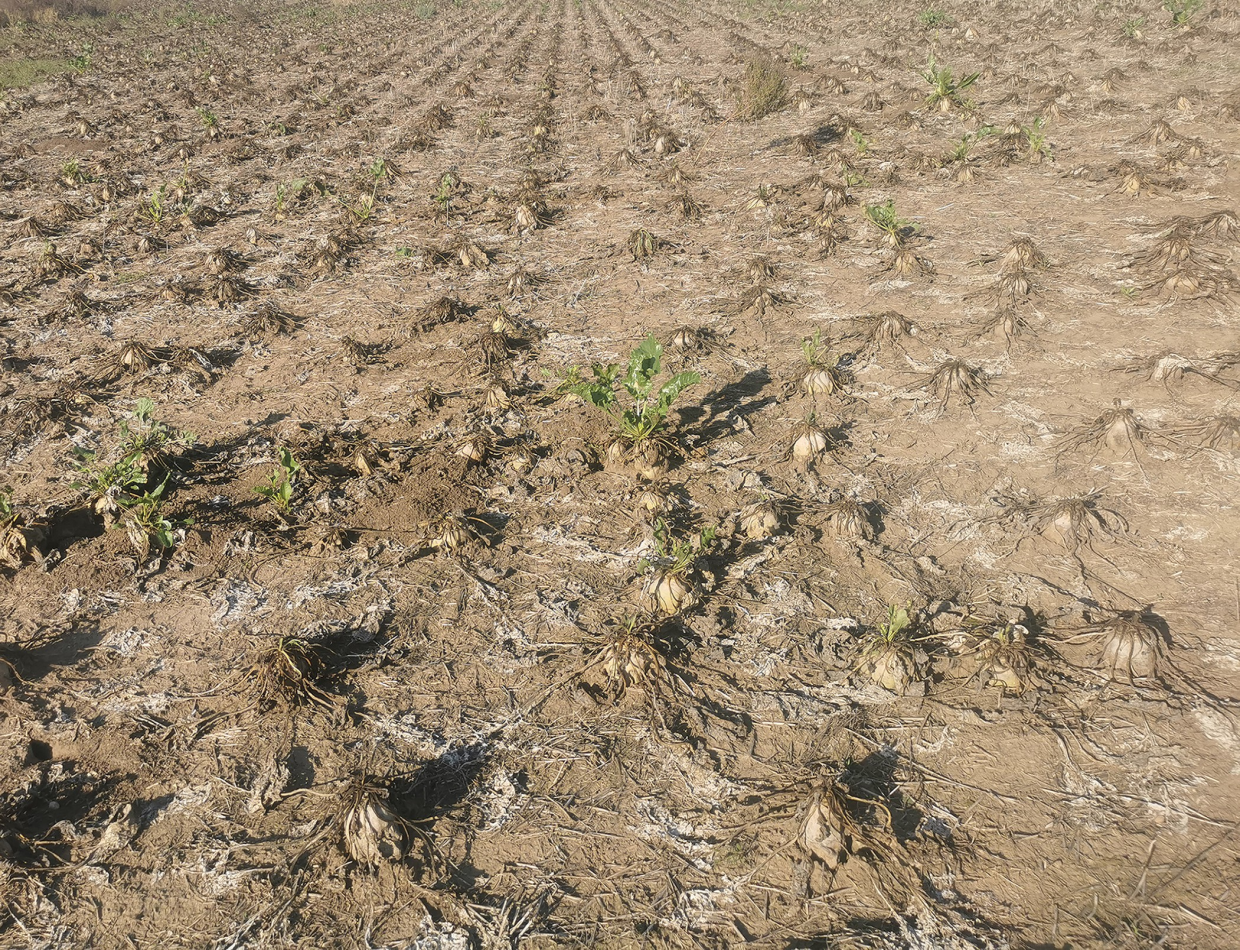
Symptoms observed on sugar beet in Kamenín, Slovakia. The completely declined and abandoned sugar beet field with eventually plowed unharvested field.

Typical symptoms associated with infection by fastidious phloem-limited pathogens (RTD/SBR symptoms) were observed in sugar beet across production fields in all surveyed countries, though not uniformly in all regions. Notably, no single sugar beet with specific RTD/SBR symptoms was found in any of the eight localities in five areas in Bohemia (CZ) despite thorough surveying. Nevertheless, samples exhibiting aspecific symptoms, which could be attributed to phytoplasma/proteobacterium infection, as well as other diseases or disorders, were collected from four localities—Jizerní Vtelno, Židněves, Konecchlumí and Chrudim. Meanwhile, no symptoms were observed in the remaining four localities in Bohemia, thus no samples were collected there. Similarly, in 31 surveyed areas in Poland, specific RTD/SBR symptoms were present only in Węgierskie with a prevalence of ~5% in the most severely affected part of the field. Aspecific symptoms were present in six localities (Piotrowice Świdnickie, Żołędnica, Chwałów, Rawicz, Babin and Buk), while no symptoms were observed in the remaining 24 localities, where no samples were collected. Aspecific symptoms encompassed proliferation of younger leaves, leaf yellowing and necrosis. In all other evaluated regions, typical RTD/SBR symptoms were observed with varying prevalence. In the Pannonian plain, sugar beets with typical RTD/SBR symptoms were found in all assessed areas, though with varying prevalence. In Moravia (CZ), typical RTD/SBR symptoms were observed in the locality of Prušánky, in epidemic scale ranging from ~10% on one side of the field to ~100% on the other, whereas in all other areas (Lednice, Litobratřice and Břežany), they were present in non-epidemic scales of ~0.1% (Table 1). Similarly, RTD/SBR-symptomatic sugar beets were observed in all assessed localities in Slovakia. While the disease was present in the northern areas (Považany and Špačince) at non-epidemic scales of ~0.1%, it reached epidemic scales of over 50% in the southern areas (Sereď, Rastislavice and Kamenín) (Fig 2). Sugar beets with typical RTD/SBR symptoms were also collected in Serbia and Hungary (both in the Pannonian Plain) from all assessed areas. In Austria, sugar beets were collected from areas where the disease prevalence was ⁓50%, as well as from areas where it was present sporadically (~0.1%). However, no data concerning disease prevalence was available for Romania. The list of surveyed localities with disease prevalences (where available) and the number of collected samples are provided in Table 1.

### ’*Ca*. P. solani’ 16SrXII-A is the dominant pathogen in diseased sugar beet in Central Europe

Phytoplasmas were detected in all assessed regions, with the exception of Bohemia (CZ), where no phytoplasma was detected in any of the collected samples with aspecific symptoms. In the Pannonian Plain and Romanian Moldova, only ’*Ca*. P. solani’ 16SrXII-A was detected in sugar beet in all assessed areas. In Poland, ’*Ca*. P. solani’ 16SrXII-A was detected in three samples (Piotrowice Świdnickie), and ’*Ca*. P. solani’-related strain 16SrXII-P was detected in one sample (Żołędnica). Additionally, ’*Ca*. P. asteris’ 16SrI was detected in four samples (Węgierskie), of which three were identified as 16SrI-A and one as 16SrI-C (Table 1). Among additional samples collected outside of Central Europe, ’*Ca*. P. solani’ 16SrXII-A was detected in 19 symptomatic samples from a field in Berny-en-Santerre, France, and in three sugar beets from a clamp in Ust-Labinsk, Russia.

### Molecular characterization of phytoplasmas

A total of 93 ’*Ca*. P. solani’ 16SrXII-A-positive samples (up to three per area) were subjected to MLSA. Comparative analysis of the 16S rDNA fragment of all ’*Ca*. P. solani’ 16SrXII-A strains detected in this study (in sugar beets from seven countries), as well as strains previously characterized in Serbia, revealed that all the examined strains shared identical sequences.

Analysis of the *tuf* gene sequences revealed two tuf-types among isolates from sugar beet in Central Europe, with tuf-d being the most common, followed by tuf-b1 (Table 2). As the predominant tuf-type in the Pannonian Plain, tuf-d was the only tuf-type found in Slovakia, Hungary and Austria, while Serbia and Moravia (CZ) each had one sample that harbored the tuf-b1 genotype. Among 75 evaluated sugar beet samples from the Pannonian Plain, the tuf-d type was detected in 73 (97.3%), while tuf-b1 was found in two (2.7%). The tuf-d type was also found in three samples from Poland, identified as 16SrXII-A, while nine tuf-d and five tuf-b1 genotypes were detected in the Wallachian Plain of Romanian Moldova. Multiple sequence alignment of *tuf* gene sequences obtained from 91 isolates showed no intra-tuf-type variability among strains in Central Europe.

**Table 2:** Characterization of the ’*Ca*. P. solani’ 16SrXII-A detected in sugar beet in Central Europe (three isolates per locality where available).

Locality-Country (number of positive samples)	GPS	*Tuf*/*Stamp* genotype**
Piotrowice Świdnickie- PL (3)	50° 55’ 31’’ N 16° 27’ 50’’ E	3x Tuf-d/STOL
Prušánky-CZ (25)	48° 49’ 14’’ N 16° 59’ 47’’ E	3x Tuf-d/STOL
Lednice-CZ (15)	48° 47’ 39’’ N 16° 46’ 31’’ E	3x Tuf-d/STOL
Litobratřice-CZ (13)	48° 52’ 47’’ N 16° 25’ 47’’ E	2x Tuf-d/STOL
		1x Tuf-b1/Rqg31
Břežany-CZ (15)	48° 52’ 35’’ N 16° 22’ 27’’ E	3x Tuf-d/STOL
Sereď-SK (25)	48° 16’ 47’’ N 17° 41’ 02’’ E	3x Tuf-d/STOL
Špačince-SK (5)	48° 25’ 47’’ N 17° 34’ 22’’ E	3x Tuf-d/STOL
Považany-SK (9)	48° 42’ 38’’ N 17° 50’ 25’’ E	3x Tuf-d/STOL
Rastislavice-SK (10)	48° 08’ 57’’ N 18° 03’ 18’’ E	3x Tuf-d/STOL
Kamenín-SK (16)	47° 53’ 55’’ N 18° 36’ 19’’ E	3x Tuf-d/STOL
Tirzii, Vaslui-RO (9)	N.A.	2x Tuf-d/STOL
		1x Tuf-b1/Rqg31
Dângeni, Botoşani-RO (2)	N.A.	2x Tuf-b1/Rqg31
Pașcani, Iași-RO (3)	N.A.	2x Tuf-d/STOL
		1x Tuf-b1/Rqg31
Hănești, Botoşani-RO (3)	N.A.	2x Tuf-d/STOL
		1x Tuf-b1/Rqg31
Bumbata, Vaslui-RO (6)	N.A.	3x Tuf-d/STOL
Groβkrut-AT(8)	48° 39’ 46’’ N 16° 45’ 00’’ E	3x Tuf-d/STOL
Pamhagen 1-AT(5)	47° 42’ 29’’ N 16° 53’ 38’’ E	3x Tuf-d/STOL
Pamhagen 2-AT(10)	47° 41’ 49’’ N 16° 57’ 05’’ E	3x Tuf-d/STOL
Pamhagen 3-AT (1)	47° 41’ 27’’ N 16° 55’ 18’’ E	1x Tuf-d/STOL
Pamhagen 4-AT (9)	47° 41’ 44’’ N 16° 54’ 43’’ E	3x Tuf-d/STOL
Wallern im Burgenland-AT (10)	47° 44’ 03’’ N 16° 57’ 00’’ E	3x Tuf-d/STOL
Andau-AT (9)	47° 47’ 40’’ N 17° 00’ 02’’ E	3x Tuf-d/STOL
Hollern-AT (7)	48° 04’ 32.0’’N 16° 52’ 34.1’’E	3x Tuf-d/STOL
Sommerein-AT (2)	48° 00’ 30.0’’N 16° 38’ 11.1’’E	2x Tuf-d/STOL
Cegléd-HU (9)	47° 12’ 57’’ N 19° 43’ 55’’ E	3x Tuf-d/STOL
Nadudvar-HU (12)	47° 25’ 47’’ N 21° 11’ 42’’ E	3x Tuf-d/STOL
Szentes-HU (12)	46° 36’ 21"N 20° 24’ 36"E	3x Tuf-d/STOL
Szikancs-HU (7)	46° 21’ 57" N 20° 28’ 44" E	3x Tuf-d/STOL
Temerin-RS (37)	45° 24’ 21" N 19° 55’ 34" E	3x Tuf-d/STOL
Botoš-RS (11)	45° 19’ 53" N 20° 34’ 27" E	3x Tuf-d/STOL
Sremska Mitrovica-RS (12)	45° 01’ 43" N 19° 39’ 38" E	2x Tuf-d/STOL
		1x Tuf-b1/Rqg31
Mol-RS (4)	45° 45’ 23" N 20° 04’ 25" E	3x Tuf-d/STOL
Ust-Labinsk-RU (3)	N.A.	1x Tuf-b1/Rqg50
		1x Tuf-b1/**RU106/23**
		1x Tuf-b1/ **RU122/23**
Berny-en-Santerre-FR (19)	49° 51’ 20" N	2x Tuf-b1*/FR662/23
	02° 50’ 41" E	1x Tuf-b1/**FR665/23**

*Genotype Tuf-b1 with an SNP, identical to Z187 (MZ604974) [6]. Novel genotypes described in this study are labeled in bold

**Stamp genotypes STOL, Rqg31 and Rqg50 correspond to St4, St2 and St1, respectively [47, 48]; N.A.–Not available.

Molecular characterization of the *stamp* gene resulted in the detection of two genotypes in sugar beet (Table 2), which strictly correlated with *tuf* genotyping. The prevailing *stamp* genotype was STOL (St4), identified in all 85 samples characterized as tuf-d, while *stamp* genotype Rqg31 (St2) was found in all seven samples characterized as tuf-b1.

Molecular characterization of additional ’*Ca*. P. solani’ 16SrXII-A-positive samples from Western and Eastern Europe, specifically three each from France and Russia, confirmed conservation of the 16S rRNA gene. This gene is identical among all strains examined in this study. Analysis of *tuf* gene sequences revealed the presence of the tuf-b1 type in all samples from Russia and one from France. However, in two samples from France (represented by FR662/23), a *tuf* genotype identical to Z187 from West Germany (tuf-b1 with a single nucleotide polymorphism, SNP) was detected. Among the three loci assessed, the *stamp* gene exhibited the greatest variability, which correlated with *tuf* genotyping. Notably, two genotypes were identified in samples from France, and three genotypes were found in samples from Russia. The strains from France (FR662/23), which had a *tuf* genotype identical to Z187, harbored a novel *stamp* genotype closely related (differing by two SNPs) to genotype Z187 (MZ604974). Meanwhile, the strain with the tuf-b1 genotype harbored another novel *stamp* genotype closely related (differing by three SNPs) to genotype PO (St10) from France. One sample from Russia possessed the Rqg50 (St1) *stamp* genotype, while the other two genotypes were novel. One of these (RU122/23) is most similar to strain AZ-GR23-14 isolated from grapevine in Azerbaijan (differing only by a single SNP), while RU106/23 is most similar (differing by 15 nucleotides) to various strains found in several hosts from the Balkans, Eastern Europe and the Middle East [28] (Table 2).

**Fig 3:**
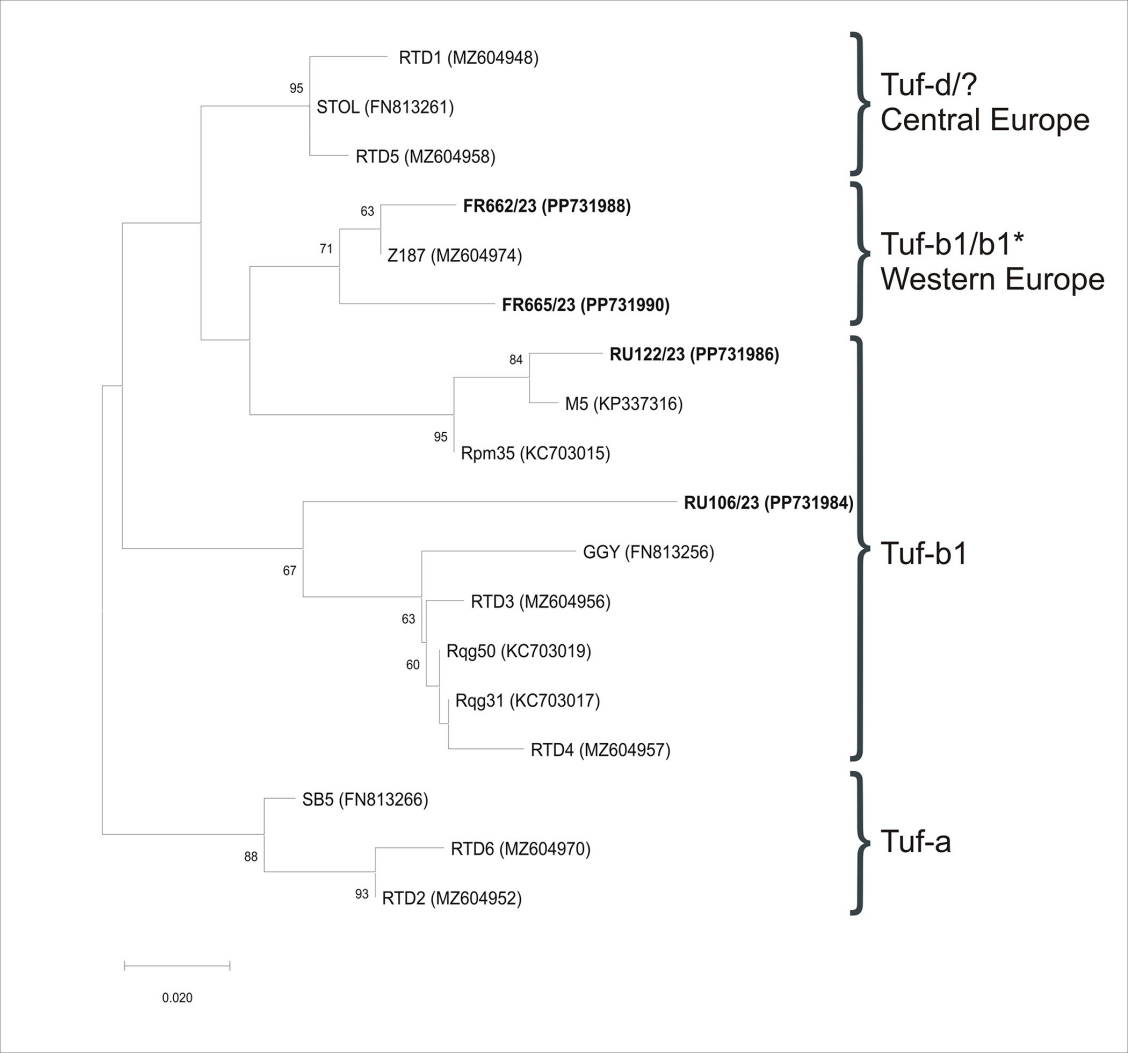
Maximum Likelihood phylogenetic analysis inferred from–*Ca*. P. solani’ *stamp* sequences obtained from sugar beet in the current and previous studies. Names of strains with novel genotypes identified in this study are labeled in bold. GenBank accession numbers are noted in parentheses. Bootstrap support values (≥60), from 1,000 replicates, are provided adjacent to branches. Scale shows substitutions per site.

An ML phylogeny, constructed from *stamp*-deduced amino acid sequences of the ’*Ca*. P. solani’ 16SrXII-A strains (obtained from sugar beet in the current and previous studies), resulted in several well-supported branches showing, to some extent, correlation with epidemiology and geographical origin. Notably, aside from a separate cluster of tuf-a epidemiology within the tuf-b epidemiology cluster, strains from Western Europe (France and West Germany) formed a separate branch. Strains from Central and Eastern Europe formed multiple branches with those bearing the tuf-d type clustered separately (Fig 3).

The phytoplasma strain detected in the sugar beet sample (PL359/23) from Żołędnica, Poland, and identified as ’*Ca*. P. solani’-related (16SrXII-P), was characterized based on sequences of 16S rDNA, *tuf*, *secY*, and *rp* genes. The sequence analysis revealed that the strain was identical to 16SrXII-P strains from East Germany, specifically strain 916/22 (accession numbers: OQ717667 for 16S rDNA, OQ717008 for *tuf*, OQ717010 for *secY*, and OQ717009 for *rp*).

### Assessment of ’*Ca*. A. phytopathogenicus’

With the exception of Poland, the presence of ’*Ca*. A. phytopathogenicus’ was confirmed in sugar beet across all evaluated countries. Compared to phytoplasmas in all surveyed countries, the abundance of ’*Ca*. A. phytopathogenicus’ was lower, especially in the Czech Republic (Moravia), Serbia, and Romania, where only two, two and one positive samples were found, respectively. The ’*Ca*. A. phytopathogenicus’-specific TaqMan qPCR yielded positive reactions (i.e. generated amplification curves had exponential growth and C_q_ values <40) for two samples in one locality of Moravia (CZ), 23 samples in three areas in Slovakia, one sample in Romania, three samples in one location in Serbia, five samples in one location in Hungary, and 33 samples in four areas of Austria. In total, 67 samples tested positive in the qPCR assay, with recorded C_q_ values ranging from 22 to >38. Samples from France and Russia tested negative in the ’*Ca*. A. phytopathogenicus’-specific TaqMan qPCR assay.

As the presence of ’*Ca*. A. phytopathogenicus’ has not been documented previously in any of the assessed countries except Hungary, we implemented two additional end-point PCR assays on qPCR-positive samples. A sample was considered positive for ’*Ca*. A. phytopathogenicus’ only if it yielded positive results in assays based on least two independent methods. End-point PCR assays revealed that the Alb1/Oliv1 primers exhibited greater sensitivity than Fra5/4, yielding positive results for 53 and 45 samples, respectively. However, the implementation of a semi-nested system enhanced the sensitivity of the Fra5/4 primer pair, aligning its performance to that of Alb1/Oliv1. Ultimately, a total of two samples from the Czech Republic, 13 samples from three areas in Slovakia, one sample from Romania, two samples from a Serbian area, five samples from a Hungarian area, and 30 samples from four areas in Austria tested positive in at least two independent assays (Table 1).

For further characterization, three ’*Ca*. A. phytopathogenicus’-positive sugar beet samples per area, which yielded amplification products of pathogen 16S rDNA using the Fra5/4 primer pair, were randomly selected and subjected to sequencing. Multiple sequence alignment of obtained sequences showed that all samples, except one from Sereď, Slovakia (SK464/23), were identical to each other and the SBR-associated ’*Ca*. A. phytopathogenicus’ type strain (AY057392), as well as to strains from Germany (represented by strain HN1220/5, MT139648), and four sequences of ’*Ca*. A. phytopathogenicus’ from Italy, which cause strawberry marginal chlorosis (SMC) (strains A5, A4, M3 and M7, DQ538375, DQ538377, DQ538378, and DQ538379, respectively) [7, 8, 49]. Notably, sample SK464/23 exhibited one SNP when compared to all other ’*Ca*. A. phytopathogenicus’ strains, as well as two SNPs in relation to an “Arsenophonus endosymbiont” of the louse fly from South Africa (MF429873). The 16S rDNA sequence of sample SK464/23 was consequently deposited in GenBank under the accession number PP716580.

### *Macrophomina phaseolina* assessment

**Fig 4:**
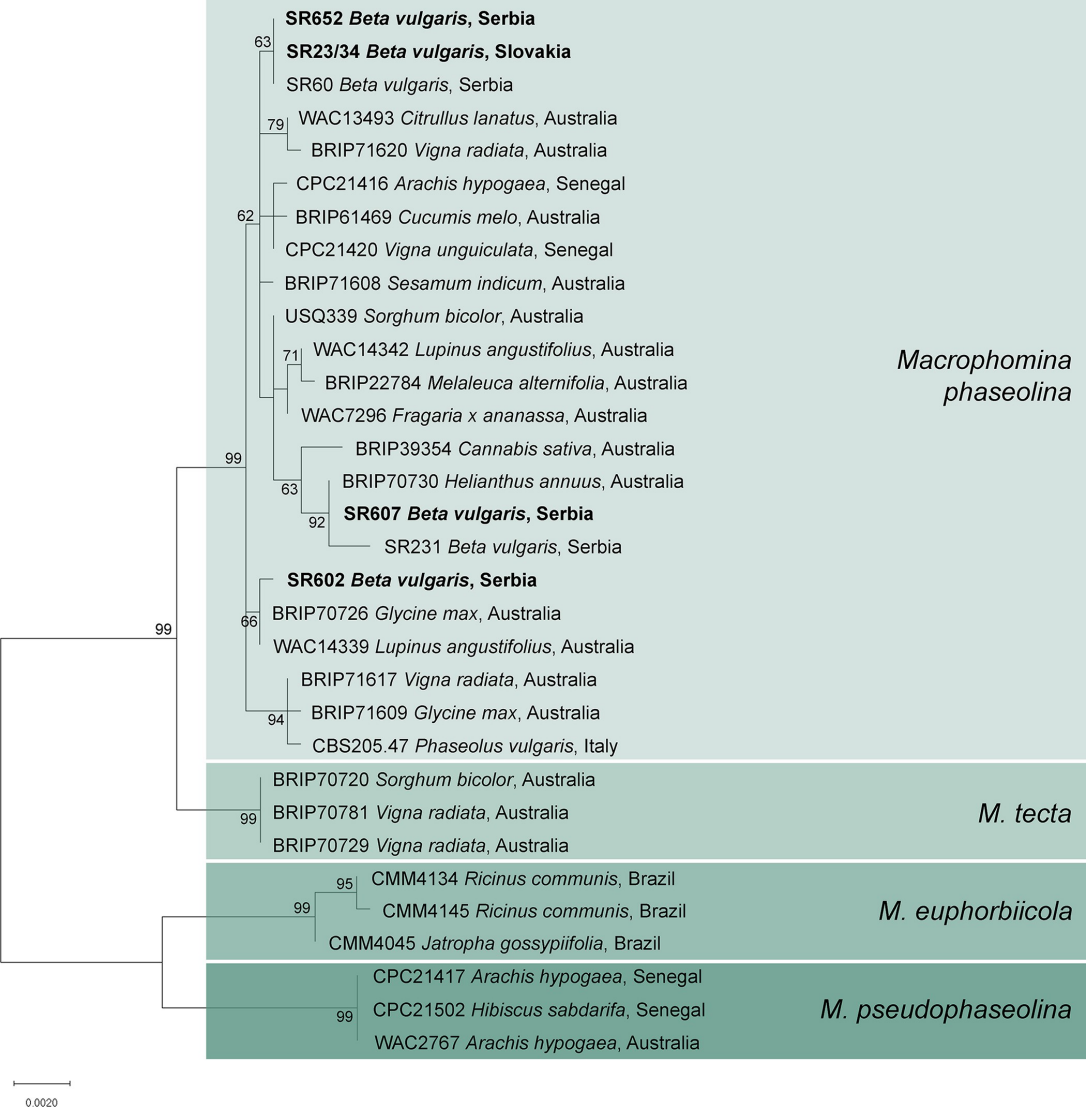
Maximum Likelihood phylogenetic analysis inferred from concatenated ITS, *TEF1*-α, *ACT*, *CAL*, and *TUB* sequences of *Macrophomina* spp. Isolates obtained in this study are shown in bold. Bootstrap support values (≥60), from 1,000 replicates, are provided adjacent to branches. Scale shows substitutions per site.

Sugar beets expressing root rot alongside RTD/SBR symptoms were detected in samples collected from the Czech Republic (Prušánky, Moravia, six plants), Slovakia (Kamenín, 12 plants), and Serbia (all four localities, 27 plants). Conversely, no evidence of root rot presence was noted in sugar beet samples from Bohemia (CZ) and Poland. Though *M*. *phaseolina* was not detected in any of the samples from the Czech Republic, *Rhizopus* sp. was isolated from four samples, while two samples contained no fungi. Fungi with dark grey colonies and numerous black sclerotia on PDA, characteristic of *M*. *phaseolina*, were isolated from eight samples from Kamenín (Slovakia) and 27 samples from Serbia (20 plants from Temerin, five from Sremska Mitrovica, and one each from Botoš and Mol). Four samples from Slovakia, however, did not contain any fungi. *M*. *phaseolina* was identified by PCR using *M*. *phaseolina*-specific primers (MpTefF/MpTefR), which amplified fragments of approximately 220 bp. All *M*. *phaseolina*-positive samples also tested positive for the presence of ’*Ca*. P. solani’-16SrXII-A in phytoplasma assessments, while ’*Ca*. A. phytopathogenicus’ analyses yielded negative results. Further characterization through *TEF1*-α sequence analyses revealed three haplotypes among isolates from Serbia (20, six, and one isolate per haplotype), and a single haplotype in Slovakia, which is identical to the dominant Serbian haplotype. Analysis of *TUB* gene sequences corroborated the three-haplotype division, while ITS, *ACT* and *CAL* were more conservative delineating only two haplotypes. In multilocus phylogeny (ITS, *TEF1*-α, *ACT*, *CAL*, and *TUB*), all isolates selected from Serbia, as well as all eight from Slovakia, formed three subclades within the *M*. *phaseolina* clade. A phylogenetic tree with representative isolates is shown in Fig 4. The currently dominant multilocus haplotype in Serbia, present in all Slovak isolates, matched the multilocus haplotype previously reported in sugar beets in Serbia [9] and clustered separately from the other multilocus haplotypes. Four other Serbian isolates had identical sequences, forming a subclade with an Australian isolate from *Helianthus annuus* (BRIP70730), and resembled the previously described Serbian isolate SR231 from sugar beet, differing only by three nucleotides in *CAL*. Isolate SR602, with a distinctive multilocus haplotype, clustered separately with isolates from *Glycine max* (BRIP70726) and *Lupinus angustifolius* (WAC14339) from Australia. The positions of isolates in the phylogenetic tree correspond to the observed differences in *TEF1*-α and *TUB* loci. In comparison to our previous study [9], two additional haplotypes were identified in the current study, resulting in a total of four *M*. *phaseolina* five-loci haplotypes detected in sugar beets in Serbia thus far.

## Discussion

As this study covers four geographically separated regions (plains) in Central Europe, obtained results may be categorized according to the plains surveyed. In the Pannonian Plain, typical symptoms associated with fastidious pathogens were found with varying incidences in sugar beet in all surveyed areas. The abundant presence of RTD symptoms in the Pannonian Plain is consistent with previous reports [6, 7, 50]. Conversely, no SBR/RTD-symptomatic sugar beets were observed in any of the surveyed areas in the Bohemian Plain (CZ). Among 31 surveyed localities in Poland (North European Plain), symptoms resembling those associated with RTD/SBR occurred with a low incidence in one locality and extremely sporadically in two others. This is consistent with previous reports of only sporadic presence of phytoplasmas on sugar beet in Poland [51, 52]. Unfortunately, there is no data concerning symptom prevalence within the Wallachian Plain in Romania.

Molecular analyses revealed the occurrence of both RTD-associated ’*Ca*. P. solani’ (16SrXII-A) and SBR-associated ’*Ca*. A. phytopathogenicus’ in Central Europe. ’*Ca*. P. solani’ 16SrXII-A emerged as the predominant fastidious pathogen and only phytoplasma present in sugar beet in all assessed countries except Poland. These fastidious pathogens were found in both single and mixed infections across all seven surveyed countries in the Pannonian, Wallachian, and North European Plains. In Bohemia (CZ), no fastidious pathogens were detected, while in Poland, only a limited presence of phytoplasmas was found, consistent with the observed symptoms in sugar beet. The detection of phytoplasmas in sugar beet in Serbia, Hungary, Slovakia and Austria (the Pannonian Plain) aligns with our previous studies [6, 7], while their presence in Romania corresponds to a historical report of RTD [50]. This study uncovered RTD-associated ’*Ca*. P. solani’ (16SrXII-A) and ’*Ca*. P. solani’-related strain 16SrXII-P for the first time in Poland. Additionally, this study represents the first report of RTD associated with ’*Ca*. P. solani’ (16SrXII-A) as an economically relevant disease of sugar beet in Moravia (CZ). In Poland, the variability among phytoplasmas (16SrI-A, -C, 16SrXII-A, -P) likely results from occasional infections due to the very low prevalence of fastidious pathogens in sugar beet. Moreover, ’*Ca*. P. solani’-related strain 16SrXII-P has been determined as the dominant fastidious pathogen of sugar beet in Eastern Germany, which shares a border and is connected by uninterrupted expanses of plain with Poland [9]. Apparently, among assessed areas, Poland is the only one where both ’*Ca*. P. solani’-related strains 16SrXII-A and -P occur, though in a sporadic manner. Furthermore, this study identifies the presence of ’*Ca*. P. asteris’ in sugar beet in Poland, consistent with its sporadic detection in Serbia and previous reports in Poland on sugar beet and other crops [6, 8, 51, 52]. Occurrence of multiple subgroups of ’*Ca*. P. asteris’ in a single field and crop is not uncommon [53, 54].

The multi-locus molecular characterization of ’*Ca*. P. solani’ in our study revealed intriguing patterns that indicate some geographical correlation. While 16S rDNA was highly conserved, with a single genotype among all analyzed sugar beet strains (in Central Europe and beyond), the two other loci (*tuf* and *stamp* genes) were more informative. Notably, we observed a strong correlation between loci genotypes and a surprising lack of variability, particularly within the **Pannonian Plain**. Among **75 analyzed samples**, tuf-d/STOL was detected in all but two, which had the tuf-b1/Rqg31 multilocus genotype. Our findings align with previous research that links the tuf-d/STOL strain to epidemic outbreaks of RTD in both Serbia and Slovakia, as well as reports of the striking dominance of tuf-d/STOL in the Pannonian Plain [6]. The overall limited genetic variability observed in the Pannonian Plain, characterized by the dominance of a single genotype, is consistent with patterns observed during epidemic outbreaks of phytoplasmas and other plant pathogens [6, 55, 56]. Furthermore, *Reptalus quinquecostatus* (Dufour, 1833) (Hemiptera, Cixiidae) has been identified as the predominant vector responsible for transmitting this strain in Serbia [6, 7, 31]. Though multiple vector species are capable of transmitting ’*Ca*. P. solani’ to sugar beet and other plants, it has been shown that particular pathogen strain–host plant–vector species combinations are primarily responsible for the development of epidemics and are thus of economic importance. This phenomenon suggests the existence of a tightly connected ecological cycle within the RTD epidemic in the Pannonian Plain. Notably, the prevalence of the tuf-d/STOL strain may indicate that *R*. *quinquecostatus* plays a crucial role in maintaining this cycle not only in Serbia, but across the region [31]. In contrast to the situation in the Pannonian Plain, where tuf-d/STOL strain dominates (97,3% prevalence), strains tuf-d/STOL and tuf-b1/Rqg31 were more balanced in the Wallachian Plain, with prevalences of 64% and 36%, respectively.

Previously reported multi-locus genotypes, as well as novel genotypes. were identified in samples collected from Eastern Europe, while two novel genotypes were found in Western Europe. Notably, tuf-b1/Rqg50 detected in a sugar beet in Russia has already been reported in sugar beet in the Pannonian Plain [6]. However, two novel *stamp* genotypes identified in sugar beets from France were closely related to the *stamp* genotype from the geographically closest sugar beet in West Germany (Z187). Moreover, the FR662/23 *stamp* genotype is coupled with an SNP tuf-b1 variant that has been described in the same sample, Z187, in West Germany. Furthermore, two novel *stamp* genotypes found in Russia exhibited the highest similarity to those found in geographically closest countries in Asia (Iran and Azerbaijan) [28]. In addition to the genetic–geographic correlation, such molecular diversity found in samples outside Central Europe may be the result of sporadic, rather than epidemic, occurrence of the pathogen. The lack of the tuf-d type in Western and Eastern Europe could either be a consequence of the absence of the vector (*R*. *quinquecostatus*) associated with the strain in the Pannonian Plain, or it could indicate that the association between the strain and the vector is a specific characteristic of Central Europe. Further investigation is needed to clarify these questions.

Another fastidious pathogen associated with SBR, ’*Ca*. A. phytopathogenicus’, was found in the Pannonian and Wallachian Plains, but not in Bohemia and Poland. Neither was it found in Russia nor France, where it has been reported as dominant over ’*Ca*. P. solani’ in sugar beet [2]. However, where observed, its prevalence was lower compared to that of ’*Ca*. P. solani’ in all localities except two in Pamhagen, Austria. Notably, ’*Ca*. A. phytopathogenicus’ has a negligible role in Serbia (two samples), the Czech Republic (two samples) and Romania (one sample), where it was detected sporadically in one location within each country. In Slovakia and Hungary, ’*Ca*. A. phytopathogenicus’ was detected at a slightly higher frequency, while in Austria it was dominant over ’*Ca*. P. solani’ in two localities. Interestingly, plant pathogenic ’*Ca*. A. phytopathogenicus’ associated with SBR and SMC, in the curent and previous studies, occurs in mixed infection or is accompanied by ’*Ca*. P. solani’ [2, 8, 57]. The qPCR assay demonstrated greater sensitivity compared to the endpoint PCR, as shown previously [12]. However, considering the lack of records that indicate the presence of ’*Ca*. A. phytopathogenicus’ in any of the assessed countries, with the exception of Hungary [5], we deemed it necessary to employ two independent detection methods in this study to confirm its presence. Moreover, endpoint PCR (Fra5/4) followed by partial 16S rRNA gene sequencing provided reliable identification and confirmation. Sequences of strains from sugar beet in the Pannonian Plain, Germany and France, and of the SMC strain from strawberry in Italy were identical, with the exception of one sample from Slovakia that differed from SBR/SMC ’*Ca*. A. phytopathogenicus’ by one SNP and from an endosymbiont of the louse fly from South Africa by two SNPs [35, 57, 58]. The highly conserved nature of 16S rDNA in the genus *Arsenophonus* has been well-documented [58]. Despite the wealth of previously [5] and newly acquired information regarding the presence of ’*Ca*. A. phytopathogenicus’ in sugar beet within Central European countries, particularly taking into account the conservation of the Fra5/4 fragment of 16S rDNA sequences of ’*Ca*. A. phytopathogenicus’, it remains challenging to establish whether the findings in this study signify the emergence of a pathogen, or occasional transmission facilitated by an unknown or scarcely present population of its known vector, *Pentastiridius leporinus* (Linnaeus, 1761). As suggested previously [59], the occasional transmission of *Arsenophonus* endosymbionts to the sap of plants by their hemipteran hosts may lead to the erratic emergence of bacteria such as ’*Ca*. A. phytopathogenicus’, detected in the Pannonian Plain. The emerging epidemics may be explained by their subsequent acquisition and transmission by a Cixiidae vector that has associated its life cycle with the infected crop. An example is the recently reported case of potato-associated *P*. *leporinus*-’*Ca*. A. phytopathogenicus’ in Germany [60]. Therefore, even with accurate identification of ’*Ca*. A. phytopathogenicus’ based on its 16S rDNA sequence, in the absence of a confirmed association of its only known vector *P*. *leporinus* or some novel vector with sugar beet, any conclusion has to be made with caution. Nevertheless, the substantial ’*Ca*. A. phytopathogenicus’ presence in the Pannonian Plain, coupled with reports in Germany regarding its association not only with SBR of sugar beet, but also with potato disease [60], supports further monitoring of this plant pathogen in Central Europe.

Our study represents the first report of *M*. *phaseolina* presence in sugar beet plants with root rot in Kamenín, Slovakia, thus supplementing available documentation on this fungus in Serbia [61]. Notably, Kamenín stands out for recording the most substantial economic losses in Slovakia, prompting the abandonment of crops in affected fields. Identification of *M*. *phaseolina* in regions afflicted with RTD, accompanied by significant economic losses leading to crop abandonment due to root rot, aligns with a prior study emphasizing its role in exacerbating losses associated with RTD alone [9]. Furthermore, our characterization of *M*. *phaseolina* isolates from sugar beet in Serbia revealed a predominant haplotype, accounting for 20 of 27 isolates, consistent with its dominance in Serbia in 2022 and its exclusive presence in eight samples from Slovakia. In Serbia, genetic variability within *M*. *phaseolina* was observed in a limited number of isolates, revealing two haplotypes in addition to those previously reported [9]. While *M*. *phaseolina* charcoal root rot has been acknowledged as a significant issue for sugar beet in Serbia since the 1990s, our findings constitute the first report highlighting the association between *M*. *phaseolina* and ’*Ca*. P. solani’ (contributing to the RTD-charcoal root rot complex) in Slovakia [6, 9, 61].

## Conclusions

The current study provides valuable insights into the geographic distribution and prevalence of fastidious pathogens and the associated fungus *M*. *phaseolina* affecting sugar beet across various regions in Central Europe. Our results reveal the predominance of **’***Ca*. P. solani**’** 16SrXII-A, with a notable absence of genetic variability, particularly in the Pannonian Plain, which has been heavily impacted by epidemics. Novel findings of ’*Ca*. A. phytopathogenicus’ reveal its expansion across the broader Pannonian and Walachian Plains, raising a dilemma regarding its emergence or occasional transmission. Additionally, *M*. *phaseolina* poses a newfound threat to sugar beet in Slovakia. The obtained results highlight the need for further investigation into the intricate dynamics of vector–pathogen(s)–plant host interactions and the ecological factors driving disease outbreaks. Such efforts are crucial for effective management of these pathogens, as well as mitigation of their impact on crop yields and economic outcomes.

## Supporting information

S1 Table Isolates of *Macrophomina* spp. used in the phylogenetic analysis. (DOCX)
